# Prosthetic Management of a Maxillofacial Defect Post‐Mucormycosis: A Case Report

**DOI:** 10.1155/crid/7658415

**Published:** 2026-04-10

**Authors:** Farzad Kazemi, Amirhossein Fathi, Pooya Saeedi, Farzad Yeganeh, Mahsa Ghorbani

**Affiliations:** ^1^ Department of Prosthodontics, School of Dentistry, Isfahan University of Medical Sciences, Isfahan, Iran, mui.ac.ir; ^2^ Department of Prosthodontics, Dental Materials Research Center, Isfahan University of Medical Sciences, Isfahan, Iran, mui.ac.ir; ^3^ Private Practice, Mashhad, Iran; ^4^ Private Practice, Isfahan, Iran; ^5^ Dental Research Center, Mashhad University of Medical Sciences, Mashhad, Iran, mums.ac.ir

**Keywords:** case report, maxillofacial prosthesis, mucormycosis, palatal obturators

## Abstract

**Introduction:**

Mucormycosis is a rare, aggressive fungal infection primarily affecting immunocompromised individuals. It can lead to significant tissue necrosis, including maxillary destruction. This case report explores the prosthetic rehabilitation of a patient with a severe maxillary defect caused by rhinocerebral mucormycosis, focusing on the challenges and effectiveness of reconstructive interventions.

**Case Report:**

A 68‐year‐old male with a history of recent COVID‐19 infection presented with facial pain, swelling, and blurred vision. Diagnosis of rhinocerebral mucormycosis was confirmed, and the patient underwent urgent surgical debridement, including left hemimaxillectomy and enucleation of the left eye. A maxillary obturator was fabricated to restore oral and nasal cavity separation, enhancing speech and swallowing. An orbital prosthesis was integrated using magnetic attachments for enhanced retention. The patient successfully adapted to the prostheses, regaining essential functions. At the 2‐year follow‐up, he reported significant improvements in both function and esthetics, with the magnetic attachments ensuring comfort and ease of use.

## 1. Introduction

Mucormycosis is a rare, aggressive opportunistic fungal infection caused by saprophytic fungi of the order Mucorales, commonly found in soil, decaying organic matter, and other environmental sources [1]. This infection primarily affects immunocompromised individuals, such as those with uncontrolled diabetes mellitus, prolonged corticosteroid use, or organ transplant recipients [2]. The spores of these fungi are typically inhaled, leading to initial colonization in the nasal passages and paranasal sinuses [3]. If untreated, mucormycosis can rapidly invade the surrounding tissues, including the orbit and brain, through direct extension or vascular invasion, resulting in thrombosis and subsequent necrosis of hard and soft tissues [4].

Maxillary necrosis due to mucormycosis is relatively rare, given the rich vascular supply of the maxilla [5]. However, when it does occur, it presents a significant clinical challenge. Maxillary involvement often leads to extensive destruction of the alveolar bone, palate, zygomatic arch, and maxillary sinus [6]. This destruction necessitates surgical debridement and resection, often leaving patients with substantial defects that impact function and esthetics [2]. Rehabilitation in such cases can be complex, requiring a multidisciplinary approach involving surgical, medical, and prosthetic interventions [7].

The increasing incidence of mucormycosis, especially in developing countries like India, has been linked to the rising prevalence of uncontrolled diabetes and other immunosuppressive conditions [7]. The combination of environmental factors, such as humidity and high temperatures, further contributes to the high incidence of this infection in these regions [8]. Early diagnosis and prompt treatment with antifungal agents like amphotericin B, combined with surgical intervention, are crucial in managing mucormycosis and improving patient outcomes [1].

This case report presents the prosthetic rehabilitation of a patient with mucormycosis involving the left maxilla and eye, resulting in significant maxillary necrosis. The use of a removable prosthesis effectively restored function and esthetics, highlighting the challenges and considerations in managing such extensive maxillary defects. Beyond its clinical complexity, this case is noteworthy because the patient survived an unusually prolonged and medically documented 7‐month coma following extensive rhinocerebral mucormycosis—an outcome rarely reported in the literature. The severity of tissue destruction, the combination of maxillary and orbital loss, and the need for an integrated maxillofacial–orbital prosthetic solution further distinguish this case.

## 2. Case Report

### 2.1. Patient Presentation

A 68‐year‐old male presented with progressive facial pain, swelling, nasal congestion, and blurred vision in the left eye. He had a history of poorly controlled hyperglycemia, with an initial HbA1c of 7.8% and persistently abnormal levels (5.8%–6.1%) despite lifestyle‐only management. Shortly before the onset of symptoms, he contracted COVID‐19 and received systemic corticosteroids, a combination that created a susceptible metabolic and immunosuppressed state for opportunistic fungal infection.

Following COVID‐19 recovery, he developed black discoloration of the palate that was initially misattributed to medication effects, delaying recognition of mucormycosis. Within days, severe left orbital involvement emerged, including pain, discoloration, proptosis, and progressive vision loss, followed by respiratory distress and a cerebrovascular event requiring ICU admission and mechanical ventilation

### 2.2. Diagnosis and Initial Management

Histopathological and microbiological evaluation confirmed rhinocerebral mucormycosis. Intravenous liposomal amphotericin B (5 mg/kg/day; 350 mg daily for this 70‐kg patient) was started immediately, with intensive renal monitoring, hydration protocols, and electrolyte management to reduce nephrotoxicity. Despite a moderate HbA1c level during hospitalization (5.9%), strict dietary control and close glucose monitoring were maintained, though insulin therapy was not required.

### 2.3. Surgical Intervention

Due to the rapidly progressing necrosis and involvement of critical craniofacial structures, a multidisciplinary team performed an urgent left hemimaxillectomy, orbital enucleation, and extensive soft tissue debridement to eliminate infected and nonviable tissues. The patient required postoperative ICU care, during which he entered a prolonged coma lasting approximately 7 months. Neurology records attributed this to a combination of severe systemic fungal infection, overwhelming inflammatory response, multiorgan stress, and a cerebrovascular event occurring during ICU admission.

Throughout this period, he received mechanical ventilation, continuous neurological monitoring (including Glasgow Coma Scale [GCS] scoring and pupillary reflex checks), and enteral nutrition. Serial CT and MRI scans were performed to track neurological changes. Upon regaining consciousness, MRI revealed a large cerebellar‐adjacent cyst, which neurosurgery elected to manage conservatively owing to its proximity to vital neural structures. Following stabilization and discharge, the patient was prescribed antihypertensive medication, vitamin supplementation, calcium, omega‐3, and low‐dose ASA (80 mg daily) for systemic support and prevent secondary complications.

### 2.4. Prosthetic Rehabilitation

**Figure 1:**
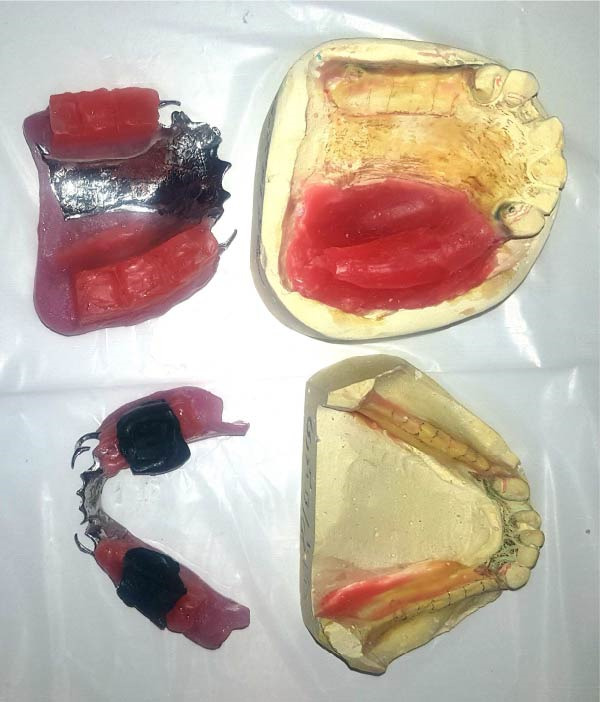
Maxillary and mandibular prosthesis base and wax rims.

The patient faced significant functional and esthetic challenges due to the extensive maxillary and orbital defects. Rehabilitation was initiated with the fabrication of a maxillary obturator to restore oral and nasal cavity separation. Initial impressions were taken with alginate (Cavex ColorChange) in a stock tray, followed by a secondary impression using a custom tray for precise defect definition. A heat‐cure acrylic base (Ivoclar) and wax rims (Polywax, Bilkim Co.) were constructed to establish vertical dimension and centric relation (Figure 1). Given the significant alteration of the maxillary anatomy, special attention was directed toward establishing an appropriate occlusal scheme. A mutually protected occlusion was selected to ensure stable bilateral contacts in centric relation while minimizing lateral interferences that could compromise the stability of the obturator.

During the wax try‐in, occlusal contacts were refined to achieve even bilateral centric contacts on the existing mandibular dentition. Excursive movements were assessed to maintain smooth working‐side guidance and eliminate non‐working‐side interferences. This occlusal strategy was implemented to enhance prosthesis stability, improve force distribution, and reduce stress on the compromised maxillary structures. Once occlusal relationships were confirmed, final fabrication and delivery were completed, resulting in marked improvement in speech, mastication, and swallowing.

Concurrently, a removable partial denture (RPD) was designed for the lower jaw to replace missing teeth and re‐establish functional occlusion. Its fabrication followed the same sequence as the obturator for continuity rehabilitation (Figure 2). During the RPD try‐in, occlusal adjustments were coordinated with the maxillary obturator to maintain balanced centric contacts and avoid premature forces capable of dislodging or destabilizing the prosthesis. This coordinated approach ensured a harmonious maxillomandibular relationship and contributed to improved chewing efficiency.

Figure 2Final maxillary and mandibular prostheses. (a) Definitive prostheses showing the maxillary fitting surface and mandibular polished surface. (b) Definitive prostheses showing the maxillary polished surface and mandibular fitting surface.

**Figure:**
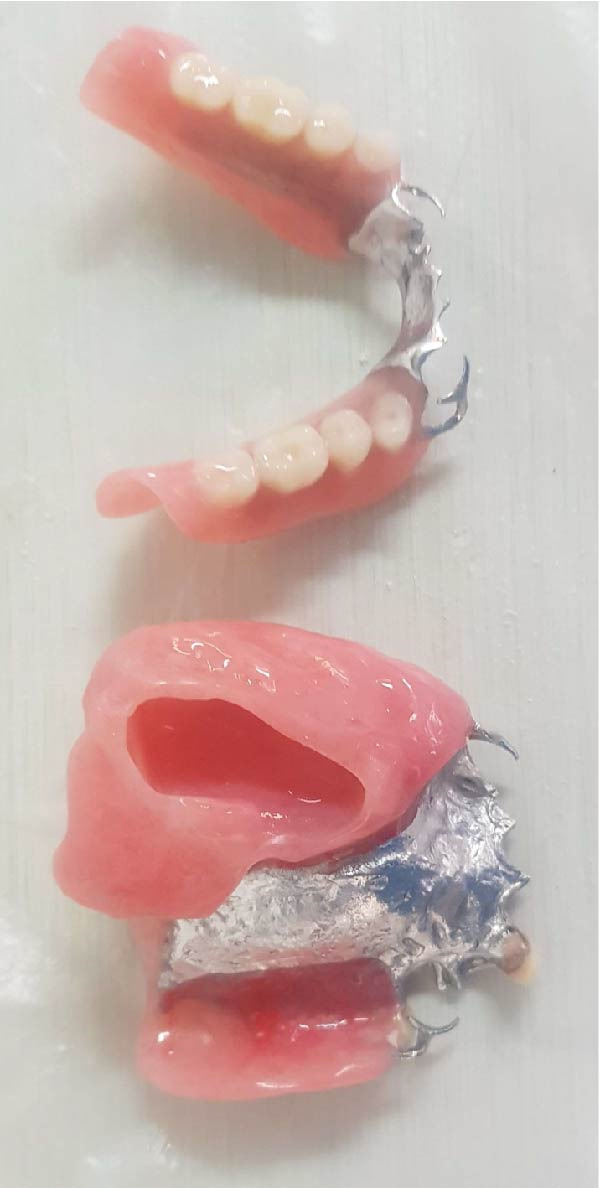
(a)

**Figure:**
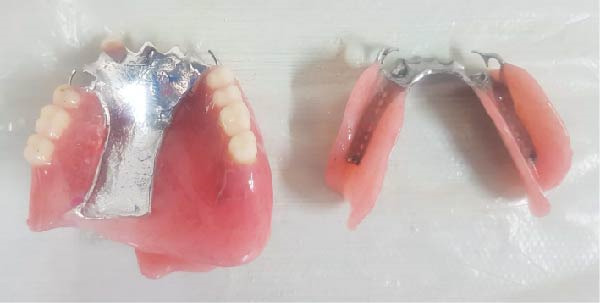
(b)

**Figure 3:**
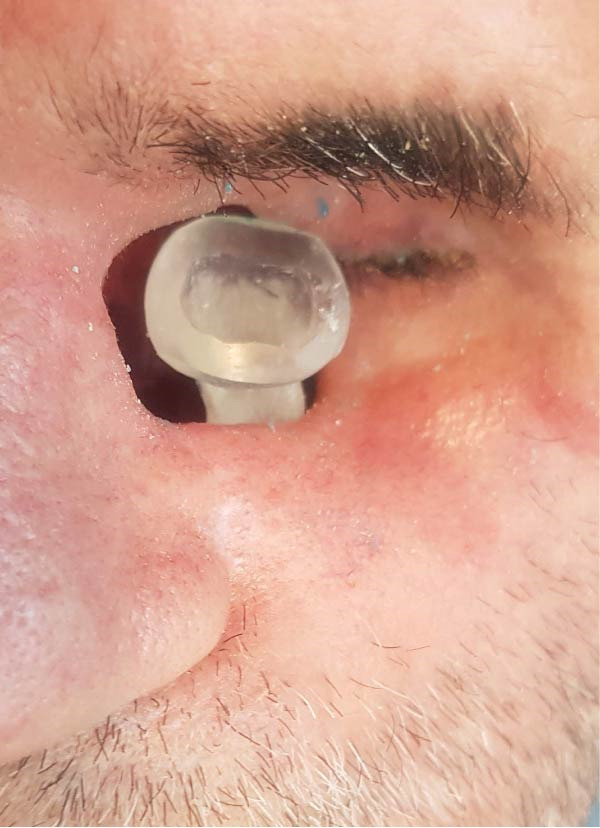
Trial fitting of the acrylic intermediary component.

For the orbital defect, a detailed approach was required to integrate an orbital prosthesis with the maxillary obturator. A wax pattern was initially designed as an intermediary attachment, fitting within the obturator’s bulb and extending into the orbital cavity. After confirming alignment, the wax pattern was replaced with self‐cure acrylic resin (Ivoclar) and processed under controlled pressure (Figure 3). The acrylic intermediary component was fabricated to host four neodymium iron boron (Ne2Fe14B) magnetic attachments (Cylandrical Nd‐Fe‐B Magnets Neomax; Hitachi Metals Ltd.): two positioned within the obturator’s bulb and two on the superior surface, interfacing with the orbital prosthesis. The prosthetic base was adjusted to prevent interference, and two additional magnets were incorporated into the tissue surface for enhanced retention. A final impression was taken using the intermediary component.

**Figure 5:**
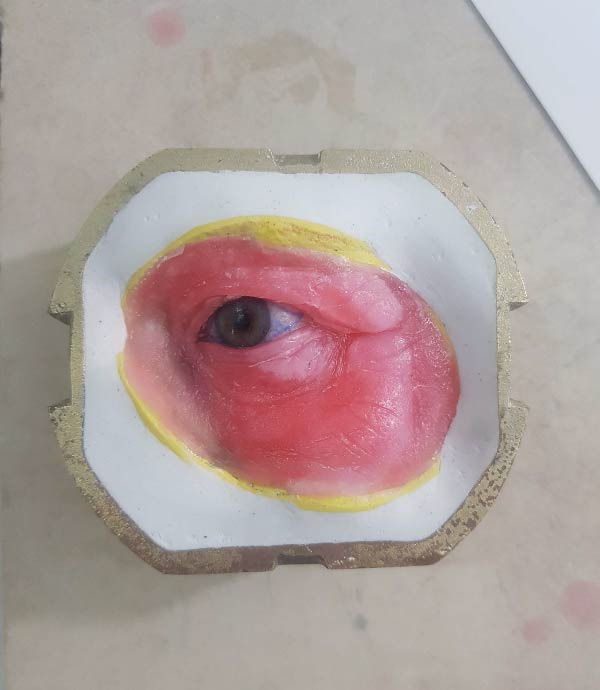
Wax pattern design of the orbital prosthesis.

**Figure 6:**
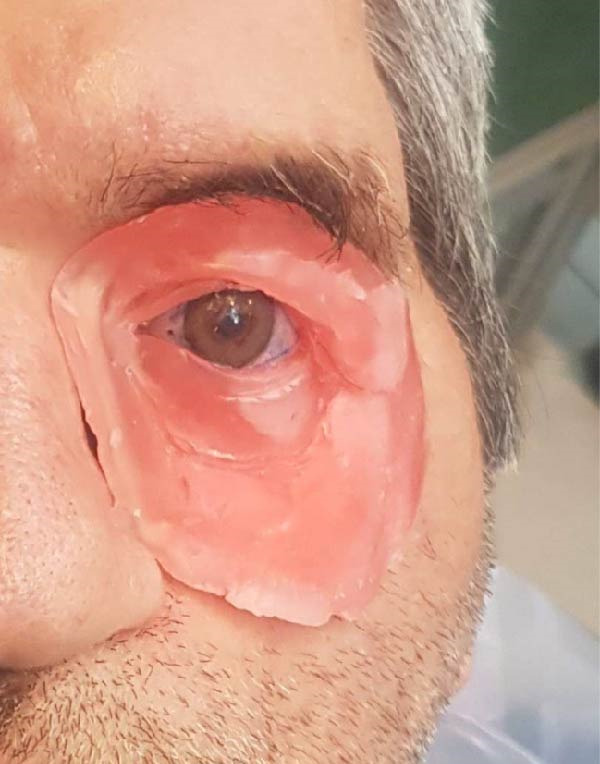
Trial fitting of the orbital wax pattern.

**Figure 8:**
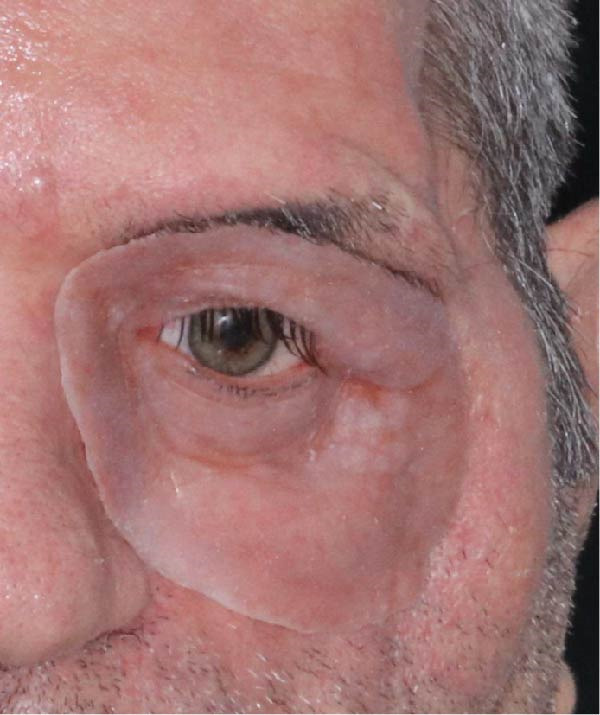
Trial fitting of the completed prostheses.

To prepare the orbital cavity for impressions, sterile gauze was used to prevent material seepage. Boxing wax (Azarteb Co.) defined the borders, and metal clasps secured the plaster (Ivorock stone) during cast‐making (Figure 4). The ocular section of the prosthesis was waxed up with close attention to symmetry and natural esthetics, referencing the unaffected eye (Figures 5 and 6). Upon approval, the prosthesis was fabricated using high‐temperature vulcanization (HTV) silicone (Technovent‐Factor 11 Inc.) to replicate tissue texture and appearance. Magnetic attachments were installed chairside for optimal retention and ease of use (Figures 7 and 8).

Figure 4Impression of the orbital component. (a) Orbital alginate impression boxed with wax and reinforced with metal retentive elements for retention of the backing material. (b) Plaster backing applied over the impression to stabilize the impression during subsequent laboratory procedures.

**Figure:**
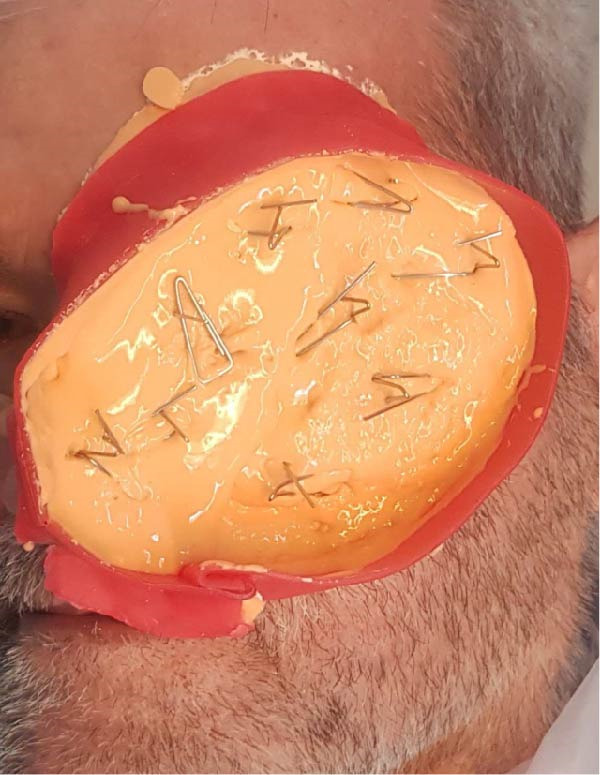
(a)

**Figure:**
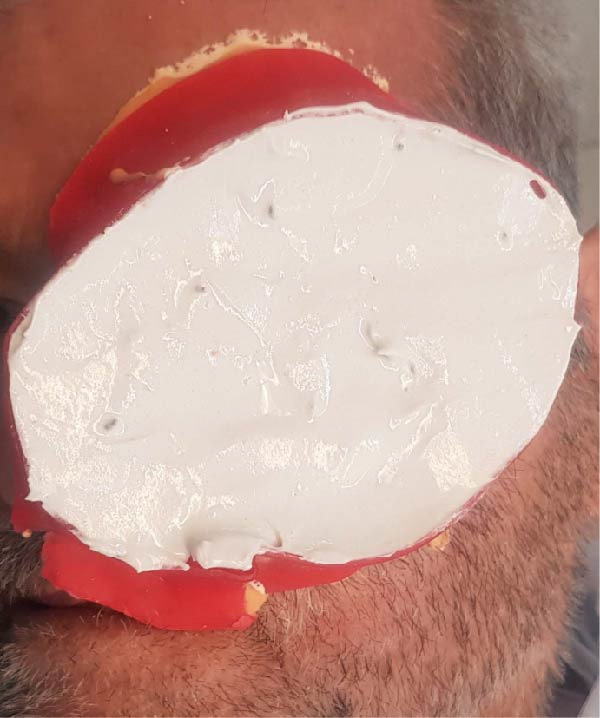
(b)

Figure 7Final fabricated prosthetic components. (a) Definitive prosthetic components after fabrication. (b) Assembly of components into the final prosthesis.

**Figure:**
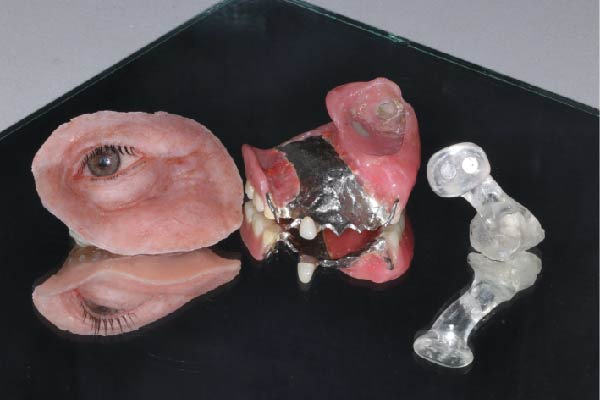
(a)

**Figure:**
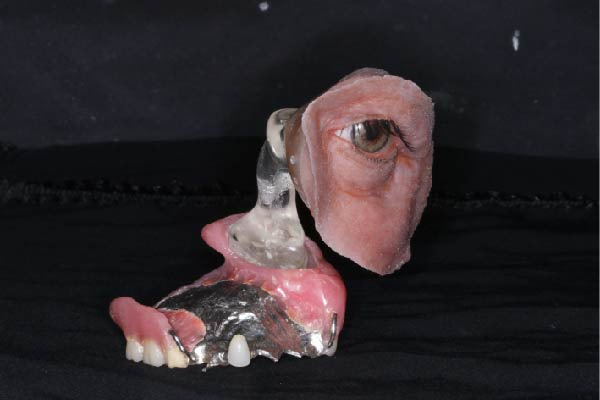
(b)

Due to the patient’s restricted mouth opening and limited cooperation following extensive maxillofacial surgery and prolonged ICU hospitalization, obtaining intraoral photographs (including maxillary occlusal views, occlusal relationships, and post‐insertion images) was not feasible.

### 2.5. Outcome and Follow‐up

The patient tolerated both the surgical and prosthetic interventions well, regaining essential functions such as eating and speaking with the maxillary obturator. The integrated maxillary and orbital prostheses significantly improved his quality of life, both functionally and esthetically. Magnetic attachments enhanced retention and facilitated easy insertion and removal, ensuring patient comfort and compliance. At the 2‐year follow‐up, the patient remained satisfied with his rehabilitation.

## 3. Discussion

Mucormycosis is a rapidly progressive opportunistic fungal infection most frequently observed in individuals with impaired immunity, particularly those with poorly controlled diabetes or recent corticosteroid exposure [9–11]. Although its angioinvasive behavior and destructive potential are well established, the present case illustrates an unusually fulminant progression, with palatal necrosis, orbital involvement, and neurological deterioration occurring within days of the initial mucosal symptoms. This accelerated clinical course aligns with previously reported post‐COVID‐19 cases but appears more aggressive than the typical trajectory described in earlier pre‐pandemic literature [12, 13].

The maxillofacial consequences of extensive surgical debridement in mucormycosis are well recognized, as maxillectomy defects compromise mastication, swallowing, speech, and overall quality of life [14]. In our patient, the size and configuration of the defect posed specific challenges, particularly due to the rapid loss of palatal and alveolar support, a pattern consistent with other rhinocerebral mucormycosis reports where early vascular thrombosis leads to widespread necrosis before diagnosis. These clinical similarities reinforce the importance of early recognition in at‐risk patients.

Prosthetic rehabilitation with an obturator remains the preferred option when surgical closure is not feasible, as it restores functional separation between the oral and nasal cavities and supports essential functions such as speech and deglutition [11]. However, retention is often problematic in extensive maxillary defects [15]. In our case, retention was further complicated by the irregular defect margins and limited remaining dentition, a scenario also emphasized in previously documented cases requiring meticulous design modifications and use of available undercuts for mechanical stability

Successful rehabilitation after extensive maxillectomy requires close, early and continuous collaboration among the surgical, prosthodontic and speech rehabilitation teams. Preoperative planning with the surgeon and prosthodontist can improve surgical margins while preserving critical anatomic landmarks and residual teeth that will later support an obturator; when prosthodontic input is delayed until after definitive resection, opportunities for tissue preservation and implant placement may be lost. Multidisciplinary planning, therefore, shortens the time to functional rehabilitation, reduces the number of interim prostheses needed, and improves long‐term outcomes for mastication and speech. Evidence from clinical series and reviews underlines the importance of early prosthodontic involvement in planning resection and reconstruction for maxillary defects [16].

The loss of a rigid bony base and variable soft‐tissue contours after hemimaxillectomy create specific challenges for impression making and prosthesis retention [17]. Accurate capture of residual undercuts, defect borders and mobile tissue requires staged or incremental impression techniques, and in many cases a custom approach to border molding and cast fabrication is necessary to obtain a functional, stable obturator. The incremental and two‐stage impression methods described in the literature help control material flow into an open sinus and improve reproduction of delicate margins, which in turn enhances adaptation and retention of the final prosthesis. Where available, implant placement in the residual alveolar crest can markedly improve retention and patient comfort, but this option requires early surgical planning and sufficient bone volume [18].

Speech function after obturation depends not only on closure of the oro‐nasal communication but on careful prosthetic design that respects phonetic principles. Collaboration with a speech pathologist during tooth set‐up, palatal contouring and evaluation of resonance improves intelligibility and patient satisfaction. Principles, such as establishing an appropriate vertical dimension, controlling anterior tooth position for labial and lingual consonant formation, and contouring palatal surfaces to guide airflow are practical measures that reduce hypernasality and improve articulation; these measures are supported by prosthodontic and speech rehabilitation literature [2]. In the absence of pre‐extraction records, stable intraoral landmarks such as the incisive papilla can assist in anterior tooth positioning to approximate natural phonetics and esthetics [19].

Material selection and margin management remain important practical considerations. Soft liners can reduce pressure on fragile tissues and improve initial comfort of an interim obturator, but they are prone to microbial colonization and may require frequent replacement [20]. Several laboratory and clinical studies have demonstrated higher rates of fungal colonization on certain soft lining materials compared with silicone or heat‐cured acrylics; this risk is particularly important in patients with a history of fungal infection and in prostheses that interface with nasal or sinus mucosa. Consequently, soft liners are best used judiciously as short‐term solutions while definitive prostheses are prepared, and strict hygiene and recall protocols should be emphasized [15, 21].

Finally, digital workflows and emerging biomaterials are increasingly relevant to obturator fabrication. CAD/CAM planning, intraoral and extraoral scanning, and additive manufacturing allow more accurate preoperative models, improved fit of immediate and definitive obturators, and can shorten laboratory and chairside time. Early reports demonstrate that 3D‐printed frameworks and digitally‐designed obturators provide predictable adaptation and can be combined with implant‐retained systems where indicated. While long‐term data are still accumulating, digital approaches offer promising avenues to enhance retention, reduce prosthesis weight, and improve reproducibility—advantages that are especially helpful in complex, large defects such as those resulting from fulminant mucormycosis [22].

Throughout rehabilitation, rigorous follow‐up is essential to monitor tissue health, adjust prosthetic borders as tissues remodel, and detect recurrence early. The prosthodontist’s role extends beyond fabrication to coordinating ongoing surveillance, maintenance, and re‐design as required; this longitudinal perspective is an important determinant of long‐term functional success and patient quality of life. Clinical series of obturator patients emphasize that frequent review in the first year and yearly long‐term follow‐up thereafter optimize outcomes and identify complications such as poor retention, tissue irritation, or prosthesis failure early [16].

## 4. Conclusion

This case highlights the severe complications of rhinocerebral mucormycosis in an immunocompromised patient. The multidisciplinary approach, including surgical intervention, antifungal therapy, and comprehensive prosthetic rehabilitation, played a crucial role in restoring the patient’s function and quality of life. The integration of maxillary and orbital prostheses with magnetic attachments provided both functional and esthetic benefits, enabling the patient to regain essential abilities such as speaking, eating, and social interaction. Long‐term follow‐up demonstrated the success of the rehabilitation process, with the patient expressing satisfaction with the prosthetic outcome.

## Author Contributions

**Farzad Kazemi**: conceptualization, clinical management, investigation. **Amirhossein Fathi**: clinical supervision, methodology, validation, resources. **Pooya Saeedi and Mahsa Ghorbani**: writing – original draft, writing – review and editing, supervision. **Farzad Yeganeh**: clinical management, project administration, validation.

## Funding

This study was self‐funded.

## Consent

The patient provided written informed consent for publication, with the condition that all photographs would be deidentifiable and cropped to protect their identity. Additionally, Figures 1–8 were approved by the patient before submission.

## Conflicts of Interest

The authors declare no conflicts of interest.

## Data Availability

The data that support the findings of this study are available from the corresponding author upon reasonable request.
